# The *Triticum Mosaic Virus* 5’ Leader Binds to Both eIF4G and eIFiso4G for Translation

**DOI:** 10.1371/journal.pone.0169602

**Published:** 2017-01-03

**Authors:** Robyn Roberts, Laura K. Mayberry, Karen S. Browning, Aurélie M. Rakotondrafara

**Affiliations:** 1 Department of Plant Pathology, University of Wisconsin-Madison, Madison, Wisconsin, United States of America; 2 Department of Molecular Biosciences, University of Texas Austin, Austin, Texas, United States of America; John Curtin School of Medical Research, AUSTRALIA

## Abstract

We recently identified a remarkably strong (739 nt-long) IRES-like element in the 5’ untranslated region (UTR) of *Triticum mosaic virus* (TriMV, *Potyviridae*). Here, we define the components of the cap-binding translation initiation complex that are required for TriMV translation. Using bio-layer interferometry and affinity capture of the native translation apparatus, we reveal that the viral translation element has a ten-fold greater affinity for the large subunit eIF4G/eIFiso4G than to the cap binding protein eIF4E/eIFiso4E. This data supports a translation mechanism that is largely dependent on eIF4G and its isoform. The binding of both scaffold isoforms requires an eight base-pair-long hairpin structure located 270 nucleotides upstream of the translation initiation site, which we have previously shown to be crucial for IRES activity. Despite a weak binding affinity to the mRNA, eIFiso4G alone or in combination with eIFiso4E supports TriMV translation in a cap-binding factor-depleted wheat germ extract. Notably, TriMV 5’ UTR-mediated translation is dependent upon eIF4A helicase activity, as the addition of the eIF4A inhibitor hippuristanol inhibits 5’ UTR-mediated translation. This inhibition is reversible with the addition of recombinant wheat eIF4A. These results and previous observations demonstrate a key role of eIF4G and eIF4A in this unique mechanism of cap-independent-translation. This work provides new insights into the lesser studied translation mechanisms of plant virus-mediated internal translation initiation.

## Introduction

For the majority of eukaryotic cellular mRNAs, translation initiation relies on the 5’ m^7^GpppG cap to recruit the necessary translation machinery. The main translation factor required for cap-dependent translation is the cap-binding complex (eIF4F), which is comprised of the smaller cap-binding protein subunit eIF4E that directly binds to the 5’ m^7^GpppG cap, the larger scaffold protein eIF4G which binds to eIF4E, and the helicase eIF4A [1]. eIF4E coordinates the attachment of the eIF4F complex to the RNA, and is recruited to the 5’ end of the mRNA via the 5’ cap [1]. Isoforms of the cap-binding proteins exist in plants, including eIFiso4G and eIFiso4E (eIFiso4F) [2]. The eIF4G/eIFiso4G isoforms share common binding motifs for the cap binding protein, eIF4A, and eIF3 in their C-terminal domains, but differ in size due to a large N-terminal truncation in eIFiso4G. Functionally, eIFiso4F strongly prefers capped RNAs with weaker structures [3–5]. Conversely, eIF4F can support translation of both capped and non-capped RNAs and can initiate translation of RNAs with stronger secondary structure. eIFiso4F is present in about 3–5 fold higher abundance compared to eIF4F [2, 6]. The cap-binding complex recruits the 43S ribosomal pre-initiation complex to the 5’ end of the mRNA via the eIF3-eIF4G interaction. Upon assembly on the mRNA, the 43S subunit scans down the mRNA in a 5’ to 3’ direction in search of the 5’ proximal AUG start codon. Scanning relies on the eIF4A DEAD-box ATPase and ATP-dependent RNA helicase to unwind the RNA structure. Once the 43S subunit reaches the 5’ proximal AUG start codon, the 60S ribosomal subunit is recruited to the complex and translation initiates [1].

Many plant viruses do not have a 5’ m^7^GpppG cap and some do not have a 3’ poly(A) tail. Although the mechanisms vary, viruses typically use their 5’ and/or 3’ untranslated regions (UTRs) to recruit the components of the translation initiation machinery. Some members of the *Luteoviridae* and *Tombusviridae* families, the umbraviruses, and satellite tobacco necrosis virus, which have been highly studied, use a 3’ cap-independent translation element (3’ CITE), located at the 3’ end of the RNA, to directly bind and deliver the translation initiation factors to the 5’ end of their uncapped RNA [7]. Such translation mechanism is dependent upon ribosomal scanning from the 5’ end of the mRNA [7]. Despite their divergent sequences and structures, all 3’ CITEs initiate translation by recruiting one of the components of the cap binding complex (eIF4F and/or eIFiso4F). For example, the 105-nt long *Barley yellow dwarf virus* (BYDV, *Luteoviridae* luteovirus) translation element binds to eIF4F (*K*_*D*_ = 37 ± 8 *nM*) and, to a lesser extent, eIFiso4F [8]. Satellite tobacco necrosis virus (STNV, necrovirus) RNA-1 relies on a 120-nt long translation enhancer domain (TED), predicted to form a long stem loop, to bind to the cap-binding complex. The TED can bind eIF4F and eIFiso4F nearly equally well, with only about a 2-fold preference for eIF4F (*K*_*D*_ = 17–30 *nM*) over eIFiso4F (*K*_*D*_ = 33–50 *nM*) [9]. The umbravirus *Pea enation mosaic virus* 2 (PEMV-2) 3’ CITE (PTE) binds to eIF4F (*K*_*D*_ = 48 ± 21 *nM*), but not eIFiso4F, via the cap-binding protein eIF4E (*K*_*D*_ = 58 ± 16 *nM*) rather than eIF4G (*K*_*D*_ = >800 *nM*) [10].

Some non-capped plant viruses rely on their 5’ UTRs for translation, including the largest family of plant-infecting viruses, the *Potyviridae*. These positive-sense and single-stranded mRNAs have a viral protein linked to their genomes at their 5’ ends (VPg) instead of a 5’ cap, and have a poly(A) tail at their 3’ ends [11]. Some reportedly drive translation initiation internally on the viral RNA, including *Tobacco etch virus* (TEV), *Turnip mosaic virus* (TuMV), *Potato virus Y* (PVY), and, recently, *Triticum mosaic virus* [12]. However, the exact requirement for the host translation machinery for most potyviruses remains poorly explored. The prototype 143-nt long TEV cap-independent translation enhancer preferentially binds to eIF4G (*K*_*D*_ = 0.10 ± 0.01 μM) over eIFiso4G (*K*_*D*_ = 2.25 ± 0.33 μM) with about a 20-fold greater affinity [13]. TEV requires eIF4G to initiate translation in a cap-binding factor depleted wheat germ extract, and competes for this factor predominantly under conditions where eIF4G is limited [14, 15].

*Triticum mosaic virus* (TriMV, *Potyviridae* poacevirus) is a wheat-infecting virus that was first described in 2008 [16] Similar to the other members of the *Potyviridae*, it contains a 10,266-nt long genomic RNA, lacks a 5’ m^7^GpppG cap structure that is likely replaced by a covalently-linked genomic protein (VPg), and is polyadenylated at its 3’ end [17]. Unlike the other members of the *Potyviridae* family, which typically have short 5’ UTRs of about 60–180 nucleotides, TriMV has an exceptionally long (739 nts) 5’ UTR [17]. This discovery heightens the remarkable diversity of translational enhancers within the family [17]. We recently demonstrated that the TriMV bears a powerful translation element within its 5’ UTR that has distinct features from other described plant viruses [18]. The TriMV 5’ UTR contains an internal ribosome entry site (IRES) that promotes strong cap-independent translation at the 13^th^ AUG codon both in wheat germ extract and in oat protoplasts [18]. Our initial data suggests that the TriMV 5’ UTR mediated translation is largely independent of eIF4E but involves eIF4G. In fact, in cap binding factor-depleted wheat germ extract, eIF4G alone is sufficient to drive translation, with no stimulatory effect conferred by eIF4E [18]. An 8-base pair long hairpin structure at nucleotide positions 469–490 is essential for internal initiation activity of the element and is a key component of TriMV 5’ UTR-mediated competition against a capped RNA *in vitro* [18]. Disrupting the stem structure by mutating the upper portion of the structure within the full-length 5’ UTR from three consecutive guanines into three cytosines at nucleotide positions 472, 473 and 474 impairs translation. The double compensatory mutation, which restores the stem structure, fully recovers its ability to compete against a capped RNA and IRES activities [18].

Here, we provide strong biochemical evidence that the TriMV IRES element functions by recruiting the components of the eIF4F complex. We demonstrate that the TriMV 5’ UTR interacts directly with both eIF4G isoforms independently of the cap-binding proteins or any other factor, and requires the hairpin structure positioned ~270 nts upstream of the initiation site for binding. Using bio-layer interferometry and affinity capture assays, we estimated that the viral translation element has a ten times greater affinity to the large subunit eIF4G/eIFiso4G than to the cap binding protein eIF4E/eIFiso4E. Functional assays reveal that the TriMV 5’ leader can use both eIF4G isoforms for translation. Additionally, we showed that the TriMV 5’ UTR-mediated translation is dependent upon the eIF4A helicase activity. These results and previous observations are consistent with a key role of eIF4G and eIF4A in this unique plant virus-mediated internal translation initiation mechanism.

## Materials and Methods

### RNA templates

The TriMV-5’ UTR (1–739), the mutants ΔstemG>C and ΔstemG = C, and 739–1 firefly luciferase reporter constructs were assembled in the cmyc-T3Luc (pA) plasmid as described in Roberts, *et al* 2015 [18]. The cmyc-T3Luc(pA) vector contains a T3 RNA polymerase promoter and a luciferase reporter gene with 63 adenines at its 3’ end. The luciferase negative control construct containing 596 bases of human β-globin sequence was assembled from a 441 nucleotide sequence ordered as a gBlock from IDT (corresponding to nucleotides 2604–3044 of GenBank DQ126325.1, with the ATG codons removed) using a two-step PCR approach. First, primers were designed to amplify the first 298 nts of this sequence with the Hind III restriction site placed on both ends of the sequence (Fwd: CAAAAGCTTGCAGGAAGAGATCCATCTAC, Rev: GTTAAGCTTATGACAGCCGTACCTGTC). The PCR product was then digested with the HindIII restriction enzyme and ligated with T4 ligase. Ligated products were gel extracted and PCR-amplified with a 5’ vector-specific primer (Fwd: GACGGTATCGATAAGCTTGCAGGAAGAGATCCATCTACATATCCC, Rev: GCGTCTTCGGCCATGGGAACACAGTTG), gel extracted post-PCR, and then cloned into the cmyc-T3Luc (pA) plasmid via In-Fusion cloning (Clontech) using the HindIII-linearized vector. The resulting product was produced from the ligation of two 298-nt products to yield a 596-nt long control.

The templates for the TriMV RNAs used in the pull-down assays (1–739 and 739–1) were generated by digesting the TriMV-containing reporter constructs with NcoI to exclude the luciferase reporter gene, leaving only the 5’ UTR sequence of interest located downstream of the T3 polymerase promoter.

The free TriMV 1–739 RNA used in the *trans*-inhibition assay were *in vitro* transcribed using a PCR-based template of the TriMV 5’ UTR sequence, with the forward primer sequence including the T7 polymerase promoter sequence at the 5’ end immediately before the viral sequence.

The capped control reporter construct used in the *trans*-inhibition assay was derived from the pLGMS2 plasmid [19], which contains an 18-nt 5’ UTR followed by a firefly luciferase gene and a 39-nt poly(A) tail at its 3’ end. BamHI was used to linearize the plasmid for *in vitro* transcription, which includes the poly(A) tail.

### *In vitro* transcription

RNAs were transcribed either from linearized plasmids or PCR-amplified templates using the T3 or T7 RNA polymerase from Life Technologies. The TriMV construct plasmids were linearized with SfcI to include a poly(A) tail at the end of the transcript. Reactions were assembled according to the Life Technologies protocol. All reporter mRNAs, unless specified, were transcribed with either a 3’-0-Me-m7G(5’)ppp(5’)G or G(5’)ppp(5’)A cap analog (New England Biolabs) at 4 mM concentrations. The ApppG cap analog increases the stability of the RNA without interfering with translation initiation and has no ability to recruit translation factors. Transcription reactions were set at 37°C for two hours, and then treated with Turbo DNase (Life Technologies) for 20 minutes at 37°C to remove the DNA template before RNA precipitation. RNA was ethanol precipitated using a 1/10 volume of 5 M ammonium acetate, pH = 5.3, and 2 x volume of 100% ethanol. Precipitated RNA was washed with 70% ethanol and resuspended in RNase-free water. RNA concentration was determined using a Nanodrop Spectrophotometer (ND-1000). Relative RNA concentration, equal sample loading, and quality were verified using an agarose gel.

RNAs used for the pull-down and the bio-layer interferometry assays were transcribed using the NcoI-digested luciferase reporter templates and internally biotinylated with biotin-UTP. The UTP concentration was reduced to 0.07 mM (from 2 mM) and 0.13 mM biotin-16-UTP (Roche) was added to the *in vitro* transcription reaction. No ApppG cap was added to the RNA.

Free RNAs used in the *trans*-inhibition assay were *in vitro* transcribed from PCR templates with primers that included the T7 polymerase promoter sequence. For both the unlabeled 1–739 and 739–1 RNAs and the biotin-UTP labeled 1–739 sequence, *in vitro* transcription was carried out as described above.

### Bio-layer interferometry

The Octet RED96 (ForteBio, Pall) was used to measure the binding affinity of eIF4G and eIFiso4G to the biotin-UTP labeled 1–739 RNA. 100 μL reaction experiments were set up in black 96-half well plates and performed at 23°C. Streptavidin biosensor tips (Pall) were hydrated in 200 μL of reaction buffer (45% supermix: 61 mM KOAc; 2.1 mM MgOAc; 5 mM DTT; 0.5 mM spermidine; 30% N’ buffer: 10% glycerol, 20 mM HEPES-KOH pH 7.6, 0.5M EDTA; 2.0M DTT; and 0.1M KCl for eIF4F, eIFiso4F, eIF4E, and eIFiso4E, or 0.2M KCl for eIF4G and eIFiso4G; and 25 μL water) for 15 minutes. After a baseline step of 1 minute, RNA was immobilized on the streptavidin biosensors at a concentration of 0.1 μg/μL for 5 minutes with rotation at 1000 rpm. A second and third baseline step of 1 minute each followed the immobilization to wash unbound RNA and allow for signal stabilization. Association was monitored by transferring the ligand biosensors to wells containing the recombinant proteins in a concentration of of 0, 200, 500, and 1000 nM were used for eIF4E and eIF4G, and 0, 200, 350, 500, and 1250 nM were used for eIFiso4E and eIFiso4G. The proteins associated with the RNA for 5 minutes, and dissociation occurred for 10 minutes in the reaction buffer. A final regeneration cycle was performed to strip the biotin-labeled RNA of protein and allow for one subsequent re-binding. The regeneration cycle consisted of 5 seconds in HCl pH 1, followed by 10 seconds in reaction buffer. Binding interactions in solution cause the optical density of the biosensor to change, resulting in a wavelength shift proportional to binding. A negative control RNA, ~600 nts in length and derived from the human hemoglobin sequence, was included to ensure binding specificity. Data were taken for each protein at the highest concentration tested (1000 nM eIF4G and eIF4E, 1250 nM eIFiso4G and eIFiso4E).

Data were analyzed using the ForteBio Data Analysis 9.0 software (S1 File). The y-axes of all steps were aligned to each step’s baseline, and curve fitting was performed to analyze the data. Curve fitting and K_D_ value determination was calculated using both the association and dissociation steps and a 1:1 partial local fitting model. The 1:1 model assumes that one analyte associates with one binding site on the surface of the biosensor. The local model computes kinetic constants for each individual curve. The partial fitting model does not assume that the dissociation will reach the pre-association baseline. The K_D_ values represent the affinity constant and thus represent an equilibrium state. The k_on_ and k_off_ rates are used to predict the equilibrium state of the interaction, and how quickly the system responds to changes in concentration of the protein. The k_on_ values represent the rate of association, or the rate at which the complex forms. The k_off_ values represent the rate of dissociation, or the rate at which the complex disassembles during equilibrium. The experiments were performed at least three times and the final K_D_ values reported are the averages of the replicates. Only fitted curves with an R^2^ value of 0.95 or greater were used in the analysis. For eIF4G, 200 nM and 500 nM concentrations were used. For eIF4E, 200 nM, 500 nM, and 1000 nM were used in the analysis. For eIFiso4G, 350 nM and 500 nM were used in the analysis, and for eIFiso4E, 350 nM, 500 nM, and 1250 nM were used. Averages of the K_D_ values at each concentration of protein were taken, and the standard deviation calculation was based on these averages.

### *In vitro* translation and hippuristanol assays

The *in vitro* translation reactions were performed using the wheat germ extract system kit (Promega) as described in Roberts, *et al* 2015 [18]. Each assay was performed in triplicate and repeated in at least three independent experiments. The reactions were set at room temperature for 45 minutes, and then stopped by placing the mixture on ice and adding 30 μL of 1x passive lysis buffer (Dual Luciferase kit, Promega) to each 10 μL reaction. 10 μL of each stopped reaction mixture was aliquoted onto a white 96-well plate, and luciferase activity was measured for 10 sec on a Centro XS^3^ LB 960 luminometer following the addition of 10 μL of luciferase assay reagent (Promega). For the biotin-labeled TriMV competition assay, luciferase activity of the capped control RNA, derived from the pLGMS2 plasmid and contains an 18-nt 5’ UTR sequence followed by the firefly luciferase reporter gene and a 39-nt poly(A) tail [18], was measured in wheat germ extract. Exogenously added RNA sequences were added to the capped RNA translation reaction at the defined concentrations.

The preparation of the depleted wheat germ extract and the subsequent translation assay were carried out as described in Roberts, *et al* 2015 [18]. Briefly, self-prepared wheat germ extract was passed over m^7^GTP sepharose resin (GE Life Sciences) to deplete the extract of the cap binding complexes (eIF4F and eIFiso4F). Depletion was confirmed by Western blot. The unbound fraction was collected, aliquoted, and stored at -80°C until use. *In vitro* translation reaction were conducted in 50 μL reaction volumes using 5 pmol of TriMV 5’ UTR RNA, 34 μM ^14^C-radiolabeled leucine (Perkin Elmer Life Science), and increasing concentrations of supplemented recombinant eIFiso4F, eIFiso4G and eIFiso4E proteins, as indicated. The reaction mixture incubated at room temperature for 30 minutes, and newly synthesized proteins were precipitated using trichloroacetic acid (TCA). The amount of incorporated ^14^C-radiolabeled leucine was measured using a scintillation counter.

For the hippuristanol assay, either the TriMV 5’ UTR firefly luciferase reporter construct or the control capped polyadenylated reporter construct with an approximately 600 nts long 5’ UTR derived from human β-globin sequence was tested. To each translation reaction, a 10 mM stock solution of hippuristanol (from the lab of Jerry Pelletier at McGill University) was diluted in 5% DMSO to the appropriate molar concentrations and added to the translation reactions. At the 0 μM concentration of hippuristanol, 5% DMSO alone was added to the reaction. Percent luciferase activity relative to no hippuristanol added for each construct was reported, and curves were fit to the data using the GraphPad Prism 5 software. For the recovery assay of the translation inhibition by 10 μM hippuristanol, 15 μM recombinant wheat eIF4A protein was added to the 10 μl wheat germ translation reaction. For the control (with no eIF4A), an equal volume of buffer (20 mM HEPES-KOH, pH 7.6, 0.1 mM EDTA, 1 mm DTT, 10% glycerol and 40 mM KCl) was added to the translation reaction.

### Recombinant protein expression and purification, and preparation of ribosome salt wash

The recombinant wheat eIF4G, eIF4E, eIFiso4G eIFiso4E and eIF4A were prepared as described in Mayberry *et al* 2007 [20]. The recombinant eIF4G, eIF4E, eIFiso4G and eIFiso4E proteins are all untagged [20]. Bradford assays were used to determine the protein concentrations. Functional activity was confirmed using competition/recovery and depleted assays with control RNAs prior to their use in the kinetics assay, pull downs, and depleted wheat germ assay.

Ribosome-associated proteins, or the ribosome salt wash (RSW) used for the pull-down assays, were isolated from wheat germ extract based on the extraction procedure from rabbit reticulocyte lysate described in Pisarev *et al* 2007 [21]. Briefly, 100 mL of Wheat Germ Extract Plus (Promega) was quick-thawed in room temperature water with occasional rotation. Pefabloc (0.5mg/mL, Roche) and six cOmplete Ultra EDTA-free protease inhibitor tablets (Roche) were added to the extract upon thawing. The suspension was centrifuged at 4°C in an SW41 Ti rotor for 4.5 hrs at 37,000 rpm. The ribosomal pellet was then suspended in Buffer A (20 mM Tris, pH 7.5, 2mM DTT, 4mM MgCl_2_, 50 mM KCl). The ribosomal proteins were washed in high salt to dissociate the proteins from the ribosomes by adding 4M KCl dropwise while continuously stirring at 4°C to a final concentration of 0.5M KCl. The suspension stirred for one hour to clarify the solution, and was then centrifuged at 4°C in an SW41 Ti rotor for 4.5 hours at 37,000 rpm. The supernatant, containing the ribosome-associated proteins, was dialyzed against 1L of Buffer D (20 mM Tris, pH 7.5, 2mM DTT, 0.1 mM EDTA, 10% glycerol, 0.1M KCl) overnight at 4°C, and then concentrated using the Amicon Ultra 10 kDa columns. Total protein concentration was determined by Bradford assay (0.7815 μg/μL).

### Pull down assays

For the pull-down assay, 10 μg (39.7 pmol) of each bait biotin-labeled RNA was mixed with 25 μL (19.57 μg) of ribosome salt wash (RSW), or 1 pmol of each of the recombinant purified wheat translation factors. A supermix (61 mM potassium acetate, 2.1 mM magnesium acetate, 5 mM DTT, and 0.5 mM spermidine) was added to a final reaction volume of 100 μL. The reactions were left at room temperature for 20 minutes, at which time the reaction mixtures were pipetted onto parafilm and UV crosslinked (254 nm wavelength) on the auto-crosslink function (Stratalinker 1800, Stratagene). These reactions were then added to a 100 μL suspension of magnetic streptavidin beads (New England Biolabs) that were washed and equilibrated in reaction buffer (25% Buffer D: 50 mM HEPES-KOH, pH 7.6, 10 mM magnesium acetate, 0.5 M KCl, 1 mg/mL heparin, 2 mM DTT; 75% supermix). The biotin-labeled RNA was allowed to bind to the streptavidin beads for 10 minutes before collecting the unbound fraction, washing the bead slurry twice in washing buffer (0.5 M NaCl, 20 mM Tris-HCl pH 7.5, 1 mM EDTA, 4 mM DTT), and washing once in low salt washing buffer (0.15 M NaCl, 20 mM Tris-HCl pH 7.5, 1 mM EDTA, 4 mM DTT). Protein was eluted off the beads by adding 15 μL of 6x SDS-PAGE loading dye to the beads and heating to 70 degrees for one minute. The flow through and washes for each sample were combined, concentrated using an Amicon Ultra 3kDa cutoff column and loaded on the gel. The interacting factors were analyzed using Western blot.

### Western blots

Rabbit polyclonal antiserum was prepared against native eIF4G, eIFiso4G, eIF4E, and eIFiso4E at the University of Texas M.D. Anderson Cancer Center, Department of Veterinary Science (Bastrop, TX) [5]. Western blot was performed using a 1:4000 dilution of eIF4G or eIF4E, and a 1:8000 antibody dilution of eIFiso4G or eIFiso4E antibody, blocked in 5% milk-TBS-T. Goat-anti-rabbit horseradish peroxidase antibody (Bio-Rad) was used at a 1:10,000 dilution and detected using the SuperSignal West Femto substrate (Thermo Fisher).

For semi-quantification of the ribosome salt wash (RSW), an increasing concentration of total protein (1.5, 2, 4, and 6 μg, determined by Bradford assay) was blotted in addition to 3 μl of wheat germ extract, which is approximately 90 ug of total protein. An equal molar concentration of eIF4G, eIFiso4G, eIF4E, and eIFiso4E recombinant proteins (0.2 pmol) were used as references for semi-quantification. Western blot was carried out as described above. Semi-quantification calculations for each factor were performed using the Bio-Rad ImageLab software and was based on band intensity relative to the corresponding 0.2 pmol loading control. The calculated relative intensity was used to determine the relative pmol concentration present in the RSW. The pmol concentration was then used to calculate the approximate the molar ratio of the proteins in the RSW. Ratios are relative to eIFiso4G (set as “1”), which was present in the highest molar concentration.

### Data analysis

Each experiment was performed in triplicate and independently repeated at least three times. Unless noted otherwise, statistical analysis (t test and two way ANOVA) was performed using GraphPad Prism 5.

## Results

### The TriMV 5’UTR RNA binds to eIF4G and eIFiso4G with greater affinity than the cap binding proteins

**Fig 1:**
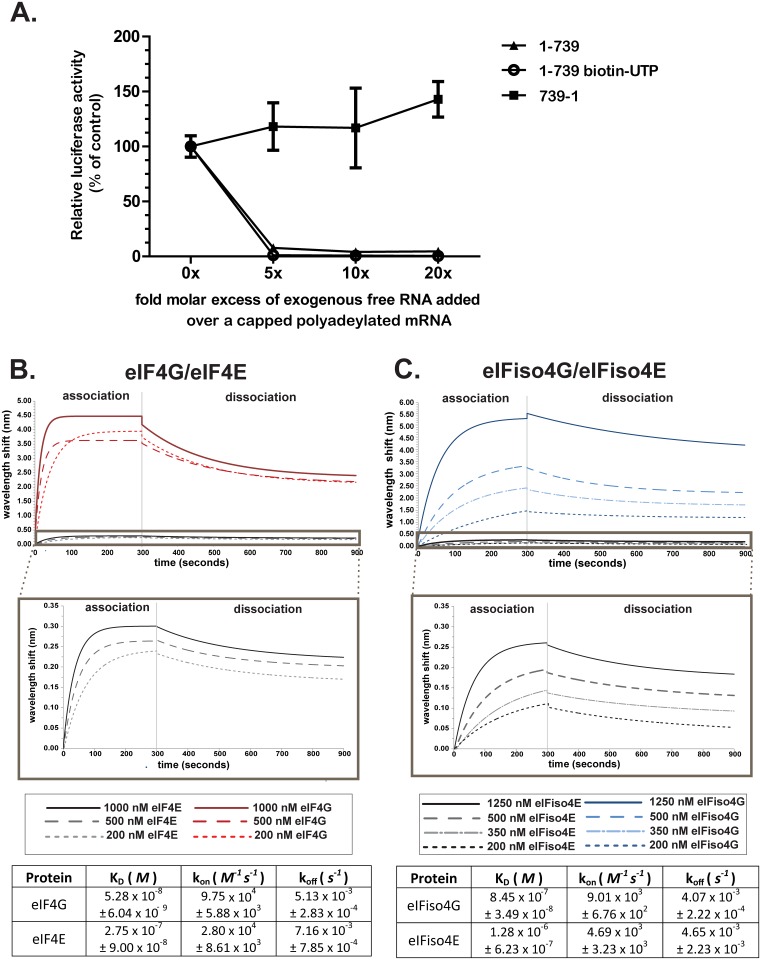
The TriMV 5’UTR RNA binds to eIF4G and eIFiso4G with greater affinity than the cap binding proteins. Bio-layer interferometry (BLI) was used to measure the binding kinetics between purified recombinant wheat eIF4G, eIFiso4G, eIF4E, or eIFiso4E protein and the viral 5’ UTR sequence (nt 1–739). Biotin-labeled RNA was bound to a streptavidin biosensor and applied to solutions containing different concentrations of proteins. A) Relative luciferase activity in wheat germ of a m7GpppG-capped and polyadenylated vector reporter in the presence of competing free RNAs in increasing molar excess A 0- to 20-fold molar excess of the competing free RNAs corresponding to the biotin-UTP labeled and unlabeled TriMV 5’UTR sequence (1–739) and the non-functional TriMV reverse sequence (739–1) were used. In B and C are shown the BLI sensograms revealing the binding curves for eIF4G/eIF4E (B) and eIFiso4G/eIFiso4E (C). A magnification of the binding curves for eIF4E and eIFiso4E is included. A table with their corresponding kinetics values is displayed below the appropriate graphs.

We previously demonstrated that the 739-nt long TriMV 5’ UTR drives cap-independent translation and supports internal initiation [18]. To show that the TriMV 5’ UTR sequence functions by recruiting the translation machinery, we measured its high-affinity interaction for each of the components of the cap-binding complex including eIF4G, eIF4E and their isoforms using Bio-Layer Interferometry (BLI). A biotin-UTP labeled TriMV 5’ UTR RNA sequence (nts 1–739) was immobilized on streptavidin-labeled biosensors. To check that the labeling of the mRNA did not impair its function, we first tested the competitive ability of the free biotinylated TriMV 5’ UTR RNA to interfere *in trans* against a capped polyadenylated reporter mRNA, presumably through sequestration of translation factors [18] (Fig 1A). We previously showed that this TriMV 5’ UTR- mediated trans-inhibition can be relieved by the addition of eIF4G [18]. We thus added free biotinylated RNA consisting of the TriMV 5’ UTR sequence (nts 1–739) at an increasing molar excess (up to 20-fold) over a capped polyadenylated luciferase reporter mRNA to the wheat germ translation reaction. The luciferase reporter construct contains vector sequence at its 5’ end and a 39 nt-poly(A) tail at its 3’ end. As controls, we used the non-labeled TriMV 5’ UTR RNA (1–739) and the TriMV reverse complementary sequence (739–1), which has no translational activity [18]. The luciferase value of the control RNA with no exogenous RNA added was defined as 100% translation (0-fold molar excess). The assay showed that the biotinylated TriMV 5’ UTR RNA interfered with cap-driven translation as strong as the unlabeled version (Fig 1A). Even at high concentrations, the non-functional TriMV reverse sequence showed no ability to inhibit translation (Fig 1A, 739–1). This confirms that the labeling of the mRNA does not interfere with its function.

We next measured the binding kinetics values (association constant k_on_, dissociation constant k_off_, and affinity constant K_D_ corresponding to the k_off_/ k_on_ ratio) with the purified non-tagged wheat eIF4G, eIF4E, eIFiso4G, eIFiso4E at increasing concentrations (0, 200, 500 or 1000 nM for eIF4G and eIF4E, and 0, 200, 350, 500, or 1250 nM for eIFiso4G and eIFiso4E) to the immobilized biotin-labeled TriMV on the Octet platform (Fig 1B and 1C). Briefly, this optical approach allows the real-time monitoring of the molecular binding events occurring on the surface of the biosensor. The binding interactions in solution cause the optical density of the biosensor to change, resulting in a wavelength shift proportional to binding. The assay was repeated in three independent experiments. Our data reveal that the TriMV 5’ UTR binds with an estimate 10-fold greater affinity to the large subunit eIF4G/eIFiso4G compared to their associated cap-binding proteins eIF4E/eIFiso4E. The TriMV 5’ UTR binding curves, with an R^2^ value of 0.95 or greater, yielded an apparent equilibrium dissociation constant (K_D_) of 5.28 x 10^−8^ ± 6.04 x 10^−9^ for eIF4G (Fig 1B), and 8.45 x 10^−7^ ± 3.49 x 10^−8^ for eIFiso4G (Fig 1C). The calculated K_D_ of eIF4E was 2.75 x 10^−7^ ± 9.00 x 10^−8^ M (Fig 1B), and 1.28 x 10^−6^ ± 6.23 x 10^−7^ M for eIFiso4E (Fig 1C). Surprisingly, we observed a statistical 10-fold difference in binding affinity of the viral RNA to the eIF4G isoforms, with a stronger affinity to eIF4G over eIFiso4G.

### The TriMV 5’ UTR binds to eIF4G in the native protein translation complex

**Fig 2:**
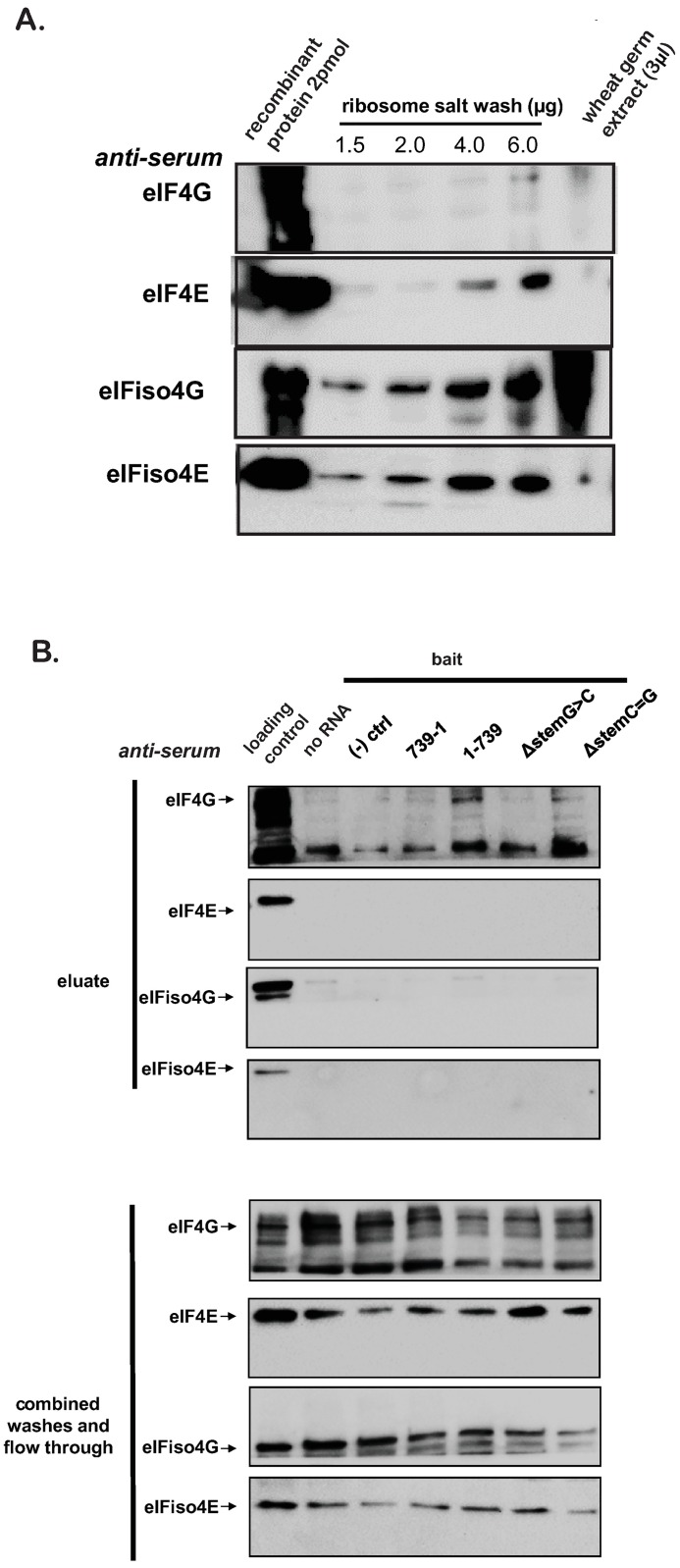
The TriMV 5’ UTR binds to eIF4G in the native protein translation complex. Biotin-UTP labeled RNAs were mixed with the ribosome salt wash (RSW) prior UV-crosslink and passed through a streptavidin column to capture the interacting factors. The TriMV 5’ UTR sequence (1–739), the TriMV reversed, non-functional sequence (739–1), a non-viral sequence control ((-) ctrl), the TriMV 5’ UTR sequence with a stem-loop structure disruption mutation at position 469–490 (ΔstemG>C), and the TriMV 5’ UTR with the double compensatory mutation (ΔstemC = G) were used as RNA baits. Western blot was used to detect each translation factor (eIF4E, eIF4G, eIFiso4E and eIFiso4G). A) Western blot against eIF4G, eIF4E, eIFiso4G and eIFiso4E of the ribosomal salt wash fractions and in wheat germ extract, relative to a fixed concentration (2 pmol) of each of the recombinant wheat factors. Using the Bio-Rad Image lab software, we approximated that eIFiso4G was present in 2.3x the concentration of eIF4G. B) Western blot against eIF4G, eIF4E, eIFiso4G and eIFiso4E proteins present in the ribosomal salt wash fraction and eluted from the biotinylated TriMV RNAs and controls. Unbound proteins obtained in the flow-through and washes are shown as well. 5 μL of RSW was added as loading control. This corresponded to 20% of the input used in the pull down.

To further assess the physical interaction of the TriMV 5’ UTR to the components of the cap binding complex, we used the biotin-labeled TriMV 5’ UTR viral sequence (1–739) as bait to pull down its interacting translation factors from an enriched fraction purified from wheat germ extract (Fig 2). This fraction, here referred to as the ribosomal salt wash (RSW), was comprised of the recovered supernatant from washing a ribosomal pellet in a high salt buffer to isolate the ribosome-associated proteins. We confirmed that the RSW was enriched with eIF4G, eIF4E, eIFiso4G and eIFiso4E (Fig 2A). The RSW maintained the approximate ratio of the factors reported in the wheat germ extract [5] with eIFiso4F being more abundant than eIF4F.

For the pull down assay, we included as negative controls the 739-nts long non functional TriMV 5’ UTR reverse complementary RNA sequence (739–1) and an approximately 600-nts long non-viral RNA sequence derived from the human β-globin sequence ((-) ctrl) (Fig 2B). We also included a control with no RNA added. The labeled RNAs were mixed with the RSW and subjected to UV-crosslinking to stabilize any RNA:protein interactions formed in the reaction prior to application to the streptavidin column. We used Western blot to detect eIF4F and its isoforms in the eluate. Using this affinity capture assay of the native translation apparatus complexes, we validated the high affinity of the TriMV 5’ UTR sequence (1–739 bait) to the large scaffold eIF4G (Fig 2B, 1–739). The viral RNA specifically cross-linked to the protein. In line with an estimated 10 fold weaker affinity of the viral RNA to its isoform (Fig 1), we failed to capture the TriMV RNA-eIFiso4G interaction above background level even if eIFiso4G was present at a high fold molar concentration comparing to eIF4G in the RSW fraction (Fig 2A). The protein was mainly detected in the washes and the flow-through. Similar observations were made for the cap binding proteins eIF4E and eIFiso4E, which were collected in the flow-through. None of the control RNAs, including the non-viral RNA ((-) ctrl) and the non-functional TriMV reverse RNA (729–1), cross-linked to any factors tested in this enriched wheat germ fraction above background level.

To correlate translation activity with factor binding, we included as bait the TriMV 5’ UTR RNA with the disruption of the 8 base-pair long stem loop at nucleotide position 469–490 within the 5’ UTR (ΔstemG>C). We previously showed that the stem structure was indispensable for IRES activity [18]. The pull-down revealed that the binding of eIF4G to the TriMV 5’ UTR required the stem loop structure. The disruption of the upper portion of the stem structure by mutating three consecutive guanines into cytosines (ΔstemG>C) eliminated eIF4G binding (Fig 2B). The double compensatory mutation (ΔstemC = G) that restored the stem structure recovered the eIF4G interaction above background level.

Taken together, our data are in line with a specific interaction of the TriMV 5’ UTR to the large scaffold protein eIF4G and a lower affinity to eIFiso4G. This interaction appears to be dependent upon a stem loop structure positioned 270 nts upstream of the initiation site.

### eIFiso4F can support TriMV 5’ UTR-mediated translation

To test whether eIFiso4G can still support TriMV 5’ UTR-mediated translation despite its lower affinity to the viral RNA when compared to eIF4G (Fig 1), we passed the wheat germ extract through an m7GpppG-sepharose column to make wheat germ extract translation dependent on exogenously added cap-binding complex. This process largely depletes the extract of eIF4F and eIFiso4F, but only a limited amount of eIF4A, eIF4B, and the poly(A) binding protein PABP due to the weaker binding interaction of these proteins to eIF4G/eIF4E and their isoforms [3]. eIF4A and eIF4B proteins are added back to the depleted wheat germ extract by default [18]. Using this system, we previously showed that eIF4F stimulated TriMV 5’ UTR-mediated translation [18].

**Fig 3:**
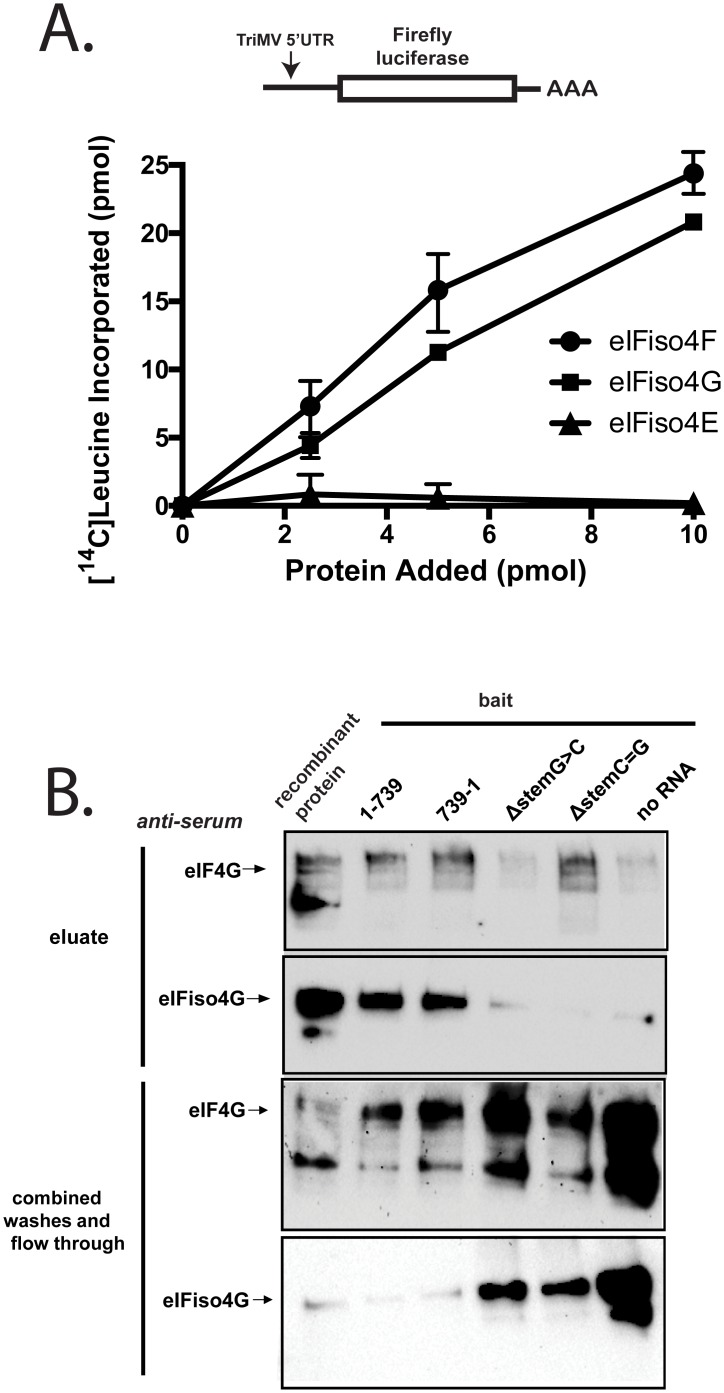
eIFiso4F can support TriMV 5’ UTR-mediated translation. A) Translation of ApppG-capped polyadenylated TriMV RNA in wheat germ extract depleted of cap-binding factors with increasing concentrations (0 to 10 pmol) of the wheat eIFiso4F complex or its individual subunits (eIFiso4G or eIFiso4E). The assay was performed with ^14^C-radiolabeled leucine, and the results display the incorporation of radiolabeled leucine (picomoles) in newly synthesized proteins following TCA protein precipitation. B) Western blot against the purified recombinant wheat eIF4G or eIFiso4G proteins individually eluated from the biotinylated TriMV RNAs and controls following UV-crosslink. Unbound proteins obtained in the flow-through and washes are shown. 0.25 pmol of each of the corresponding recombinant proteins was loaded in the first lane to serve as a ladder.

Here, we measured translation of the TriMV firefly luciferase reporter RNA in the presence of an increasing concentration (0 to 10 pmol) of recombinant wheat eIFiso4F, eIFiso4G, or eIFiso4E added to the depleted extract. The luciferase reporter construct contains the 739-nt long 5’ UTR sequence at its 5’ end and a 62-nt long poly(A) tail at its 3’ end. An ApppG cap was added to the 5’ end of the RNA. Unlike an m7GpppG cap, the ApppG cap is non-functional in translation. Translation was measured by determining the percentage of ^14^C-labeled leucine incorporated into newly synthesized luciferase proteins. Shown in Fig 3A, while eIFiso4E alone had no effect on translation, eIFiso4G alone was sufficient to drive TriMV 5’ UTR-mediated translation. The presence of eIFiso4E together with eIFiso4G (eIFiso4F) provided a slight advantage in translation compared to eIFiso4G alone (Fig 3A). This data reveal that the TriMV can use the cap binding complex isoform for its translation.

To capture the direct interaction of the TriMV 5’ UTR to eIFiso4G, we mixed the labeled TriMV RNA with purified recombinant wheat eIF4G or eIFiso4G and subjected the mixture to UV-crosslinking prior to application to the streptavidin column (Fig 3B). In the absence of any other factor when compared to the RSW assay (Fig 2), we detected the binding of the 1–739 viral sequence to both eIF4G and eIFiso4G. Under this “non-competitve” condition, the non-functional TriMV reversed RNA sequence (739–1) bound to the large subunits. However, it was clear that the interaction of eIF4G and eIFiso4G to the functional viral RNA was dependent upon the stem-loop structure at position 469–490. The mutation of the stem-loop (ΔstemG>C) failed to pull down eIF4G or eIFiso4G. However, restoration of the stem structure (ΔstemC = G construct) was sufficient to recover eIF4G but not eIFiso4G binding.

Together, these observations support that the ability of the TriMV viral sequence to bind eIFiso4F correlates with its ability to use the complex for its translation.

### TriMV requires eIF4A helicase activity for its translation

The eIF4F complex is also comprised of the DEAD-box RNA helicase eIF4A. We therefore tested the requirement for eIF4A in the TriMV 5’UTR-driven translation reaction using a functional assay. We measured translation of the TriMV luciferase reporter RNA in the presence of an increasing concentration of hippuristanol. This small molecule drug specifically inhibits eIF4A activity and has previously been used to classify eIF4A-dependent and eIF4A-independent methods of translation initiation, at least in animal systems [22].

**Fig 4:**
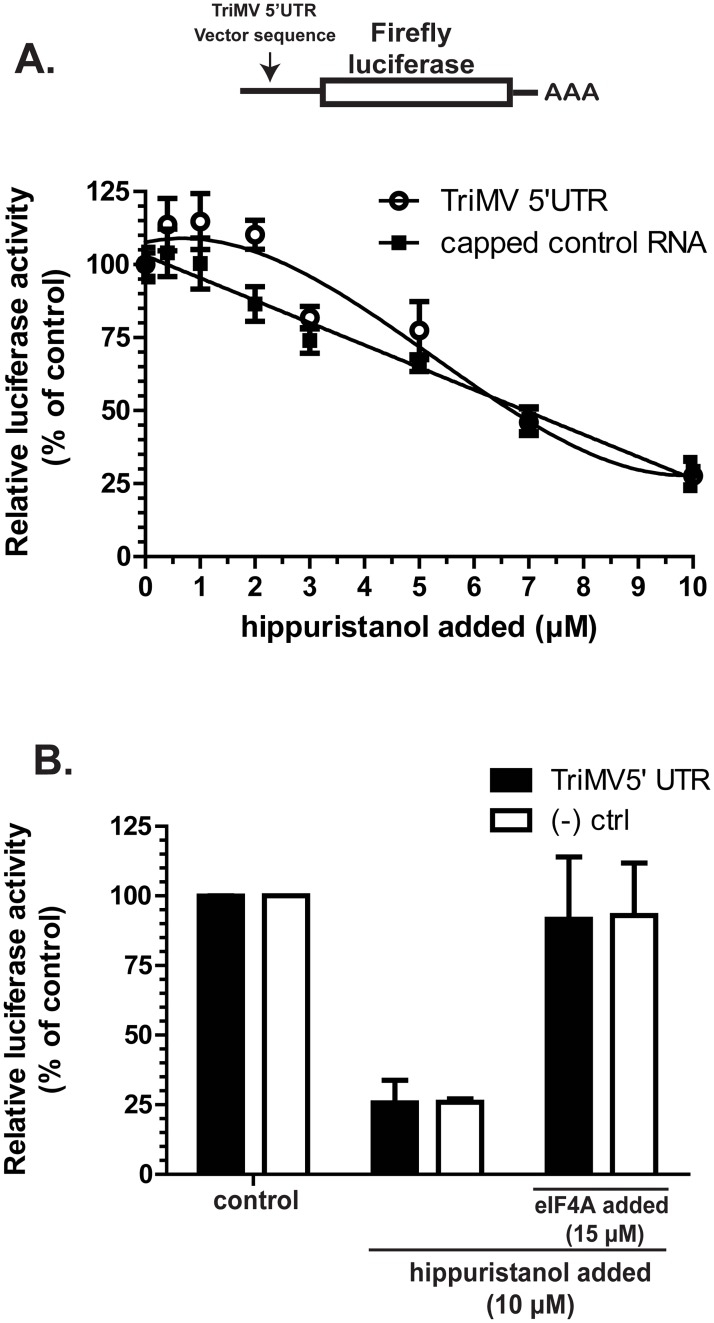
TriMV requires eIF4A helicase activity for its translation. A) Relative luciferase activity in wheat germ extract of the TriMV 5’ UTR and m7GpppG-capped control polyadenylated mRNAs with increasing concentrations of the eIF4A-inhibiting drug hippuristanol, suspended in 5% DMSO and added to the translation reaction. The luciferase activities were standardized to the measured luciferase activities of each mRNA with no hippuristanol added. At the 0 μM concentration of hippuristanol, equal volume of 5% DMSO alone also was added to the reaction. B) Translation inhibition by 10 μM hippuristanol can be reversed by adding 15 μM recombinant wheat eIF4A protein.

We compared eIF4A translation dependency of our TriMV reporter RNA construct to a capped polyadenylated control reporter RNA. The control RNA contains a 5’ UTR sequence approximately 600-nt in length, derived from the human β-globin sequence, with a 42% GC content and free energy (ΔG) value of -138.42 kcal/mol, which are similar to the TriMV 5’ UTR [18]. Our data showed that the hippuristanol was active in wheat germ extract. The increasing concentration of the drug inhibited the translation of the capped control RNA (Fig 4A). At lower concentrations, the TriMV reporter mRNA seemed to be less sensitive to the addition of the hippuristanol when compared to the capped mRNA. However, there was no significant statistical difference at most concentrations except for 2 μM (Fig 4A). Approximately 50% of translation activity was lost with the addition of 7 μM of hippuristanol for both the capped control RNA and the TriMV 5’ UTR report mRNA.

To show that the hippuristanol specifically inhibits plant eIF4A activity, we added recombinant wheat eIF4A protein to the inhibited reaction. We relieved the inhibitory effect of 10 μM hippuristanol with the addition of 15 μM of wheat recombinant eIF4A protein, which restored translation of both the TriMV reporter mRNA and the control mRNA to 100% of the control (no hippuristanol added) (Fig 4B). We conclude that the TriMV translation activity relies on eIF4A activity for translation.

## Discussion and Conclusions

These and previous results provide the first biochemical evidence that the 739 nt long TriMV 5’ UTR binds to both wheat cap binding complex isoforms strongly via eIF4G/eIFiso4G to drive cap-independent translation. First, using bio-layer interferometry and affinity capture assays, we demonstrated that strong binding of eIF4G/eIFiso4G to the mRNA occurs in the absence of the cap-binding factor eIF4E or any other factor (Figs 1 and 2). The binding interaction data suggest that the large subunit is sufficient for the IRES function of the TriMV 5’ UTR, similar to the requirement for translation of the closely-related animal picornaviruses. In picornaviral translation, the C-terminal region of eIF4G, which lacks eIF4E and PABP binding sites following viral proteinase-mediated cleavage, is sufficient to facilitate the binding of the ribosomal subunit onto their IRES elements [23–25]. It is unclear whether plant-infecting potyviruses are capable of inducing cleavage of eIF4G, which disfavors cap-dependent translation and thereby enhances the competitiveness of the virus in recruiting translation factors. However, the ability of both eIF4G and eIFiso4G to directly binding (Figs 1 and 2) and to mediate translation largely independently of the cap-binding factor (Fig 3 and [18]) may already contribute to the competitiveness of the TriMV RNA. However, we cannot dismiss the weaker but measurable affinity of the mRNA to eIF4E/eIFiso4E. It is possible that such interaction could still play a role in the assembly and/or stability of the translation machinery onto the mRNA that we were not able to capture in our assays, or as a decoy to sequester some factors away from the host mRNAs.

While the exact sequence/structure requirement for eIF4G/eIFiso4G binding on the TriMV 5’ UTR has yet to be dissected, it is clear that its binding to the viral sequence (Figs 2 and 3) is dependent upon the 8-base pair hairpin positioned about 270 bases upstream from the initiation site. The three base pair mutation within the stem that disrupts the structure was sufficient to abolish IRES activity of the viral 5’ UTR [18], which correlates with the loss of binding to eIF4G/eIFiso4G (Figs 2 and 3). Aside from direct factor binding, this structure could be involved in maintaining the general structure of the 5’ UTR for the stabilization of the cap-binding complex onto the mRNA. Interestingly, the cap binding complex isoforms show differential interaction to the hairpin (Fig 3). While the disruption of the stem structure abolished all interaction, its restoration with the double compensatory mutation restored eIF4G but not eIFiso4G binding (Fig 3B). This is in line with a potential distinct mechanism of recruitment of eIFiso4F in translation.

It is worth noting that under “non-competitive condition” we detected eIF4G/eIFiso4G binding to the reversed, non-functional TriMV 5’ UTR sequence with the purified individual factors (Fig 3), but not in the context of the native translation apparatus complex (Fig 4). Using RNA folding prediction software (mFold), we predict that the reverse complementary sequence bears also a mirror 8 nt stem loop structure with a UGGU terminal tetraloop (5’ AGAACCCUUGGUGGGGUUGU 3’, underlined the stem portion) as the one at position 469–490 in the correct orientation (5’ UCUUGGGGUGGUUCCCAAGA 3’). This could explain the observed binding. While necessary, the hairpin structure may not be the only structural/sequence requirement of the mRNA activity. We previously showed that the ability of the reverse complementary sequence to interact with the scaffold protein is not sufficient to drive translation [18]. And, as shown in Fig 1A, this RNA failed to inhibit cap-dependent translation when added *in trans*.

IRESes often use only a subset of canonical eIFs and rely on non-canonical interactions with the components of the translation apparatus [1]. Here, we revealed the need for the DEAD box RNA helicase eIF4A in TriMV 5’ UTR-driven translation. The eIF4A RNA helicase is required in cap-dependent translation, which promotes the attachment of the ribosomal subunit to the mRNA and aids with the 5’ to 3’ RNA scanning of the ribosomal subunit by unwinding RNA structure in the 5’ UTR. The requirement of the eIF4A of a particular mRNA is partially determined by the level of RNA structure present in the 5’ end of the mRNA. It has been suggested that IRES structures often need “remodeling” by eIF4A before they are able to function, as observed for the EMCV IRES [26]. A similar requirement may be occurring for the TriMV- 5’ UTR mediated translation with its sequence with a high G/C content (42%) and predicted free energy (ΔG) of -205.7 kcal/mol [17]. The eIF4G subunit in complex with eIF4A is proposed to prepare the EMCV IRES region flanking the initiation codon so that the 43S ribosomal subunit can bind productively [26].

The translation mechanism for plant viruses harboring internal ribosome entry sites is poorly understood, but it is becoming evident that plant viruses use very diverse translation mechanisms, even within their own viral families. Future studies that reveal the biological relevance of isoform flexibility could provide more insights into the viral life cycle.

## Supporting Information

S1 File Raw values of the bio-layer interferometry assays. The raw data were collected for the protein/RNA binding experiments and used for the determination of the curve fitting and Kd, Kon and Koff values in Fig 1.
(XLSX)
